# Acute bilateral simultaneous angle closure glaucoma after topiramate administration: a case report

**DOI:** 10.1186/1752-1947-2-1

**Published:** 2008-01-08

**Authors:** Kakarla V Chalam, Tina Tillis, Farhana Syed, Swati Agarwal, Vikram S Brar

**Affiliations:** 1 Department of Ophthalmology, University of Florida-Jacksonville. Jacksonville, USA

## Abstract

**Introduction:**

A case of severe acute bilateral angle closure glaucoma with complete visual loss after oral topiramate therapy.

**Case presentation:**

A 34 year-old woman developed bilateral severe visual loss 2 days after doubling the dosage of topiramate. Her best-corrected visual acuity (BCVA) was counting fingers in both eyes (OU). Intraocular pressures were 49 mm and 51 mm of Hg in right and left eyes respectively, with conjunctival chemosis, corneal edema, shallow anterior chamber and closed angles on gonioscopy. B-scan ultrasound revealed annular peripheral choroidal effusions in both eyes.

**Conclusion:**

Intraocular pressures and anterior chamber depth were normalized after discontinuation of topiramate and initiation of antiglaucoma therapy. Two weeks later, visual acuities improved to 20/25 in the right eye and 20/40 in the left eye. B-scan ultrasound showed resolution of choroidal effusion. Topiramate, an oral sulpha-derivative medication is known to cause ciliochoroidal effusions, which lead to forward rotation of the ciliary body and displacement of the lens-iris diaphragm, with resultant acute angle closure glaucoma and myopic shift.

## Introduction

Banta et al [1] reported the first case of topiramate (Topomax; Ortho-McNeil) induced acute-angle closure glaucoma in a 51-year-old man who recently initiated the medication for mood-stabilization. Topiramate, a sulfamate derivative, is primarily used in the management of seizure disorders, however has also demonstrated efficacy in the treatment of bipolar disease and migraine. In the case of migraine, the symptoms of elevated intraocular pressure secondary to angle closure may mimic those of the primary condition, thus it is important to be aware of this association as the symptoms typically resolve with cessation of the medication and management of the intraocular pressure. We present a case of bilateral acute angle closure glaucoma occurring within 10 days of initiating therapy with topiramate for symptoms related to migraine.

## Case presentation

**Figure 1:**
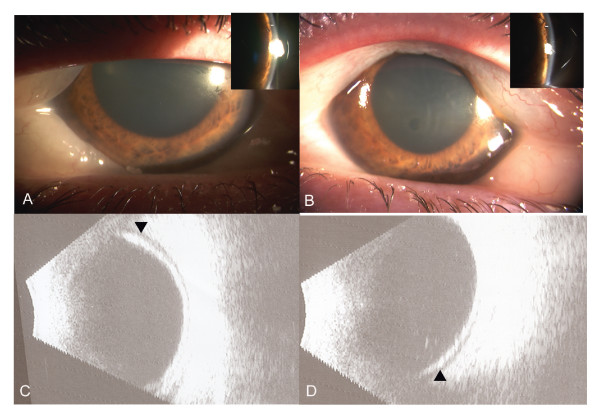
Slit-lamp photograph at presentation, revealing conjunctival chemosis, corneal edema and markedly shallow anterior chamber in right (A) and left eye (B). Insets: Slit-image showing shallow peripheral anterior chamber; depth is marked with line. B-scan ultrasound at presentation showed peripheral choroidal effusions (arrow) in Right (C) and left (D) eyes.

A 34-year-old woman with migraine, hypertension and hypothyroidism presented with acute bilateral severe visual loss after increasing her dose of topiramate from 50 mg to 100 mg daily. She had initiated the medication 1 week prior, however, her symptoms did not respond to the initial dose. Her other medications were levothyroxine, methyclothiazide, and triamterene. Best corrected visual acuity was counting fingers OU. Anterior segment examination demonstrated bilateral conjunctival chemosis, corneal edema, markedly shallow anterior chamber, and closed angles on gonioscopy. Intraocular pressures (IOP) were elevated in both eyes at 49 mmHg and 51 mmHg respectively. Peripheral iridotomy was performed on both eyes due to suspected papillary block. B-scan ultrasound revealed annular (360°) peripheral choroidal effusions (Figure 1). Topiramate was discontinued and topical Cosopt (Merck, timolol + dorzolamide); Alphagan P (Allergan, brimonidine 0.1%) and Maxidex (Alcon, dexamethasone) were started. On follow up the next day, the anterior chamber remained shallow; however the IOP had improved to 24 mmHg and 18 mmHg respectively. Deepening of the anterior chamber was noted following dilation of the pupil.

**Figure 2:**
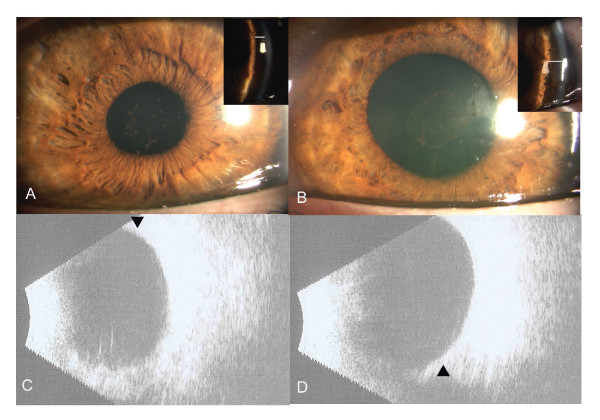
Slit-lamp photograph at day 5, revealing deep anterior chamber with resolution of corneal edema and conjunctival chemosis in right (A) and Left (B) eyes. Insets: Slit-image showing deep peripheral anterior chamber, depth is marked with line. B) B-scan ultrasound at 2 weeks shows resolution of choroidal effusions (arrow) in Right (C) and left (D) eyes.

On day five, visual acuity improved to 20/400 with resolution of the conjunctival chemosis and corneal edema. Intraocular pressures were controlled and the anterior chamber was deep (Figure 2A, B). Fundus examination showed cup to disc ratio of 0.1 in both eyes and was otherwise unremarkable. Ultrasound demonstrated mild residual choroidal effusion in both eyes which resolved two weeks later. At this point, the BCVA were 20/25 OD and 20/40 OS with the intraocular pressures and anterior chamber depth remaining stable (Figure 2C, D). Topical aqueous suppressants were discontinued.

## Conclusion

Topiramate was started for treatment of migraine. As the patient's symptoms did not respond, her neurologist increased the dosage two days prior to presentation. Patient's symptoms worsened with sudden decrease in vision and extreme ocular pain. Presence of choroidal effusion suggests an association between topiramate induced forward rotation of the ciliary process and forward displacement of the lens iris diaphragm which contributed to myopic shift, anterior chamber shallowing and resultant angle closure glaucoma. Though the exact mechanism is not clear, the fluid movement in choroidal effusion is related to drug induced changes in membrane potential. [1-5]

Topiramate is an oral sulfamate medication used primarily for epilepsy and migraine. [6] Other uses of topiramate include use in management of peripheral neuropathies [7] and radiculopathies [8], idiopathic intracranial hypertension [9], adjunctive therapy in alcohol dependence [10] and nicotine cessation. [11] There have been several reports of topiramate associated angle closure and myopic shift. Most cases have been reported to occur within 2 weeks of starting topiramate or within hours of doubling the doses. [1-3]

In our case, the neurologist doubled the dose of topiramate to alleviate non-responding headache. In retrospect, these symptoms may have been related to elevated IOP and not migraine as evident on tonometry and B-scan ultrasonography. Therefore, increased awareness is needed before initiating or modifying topiramate therapy as persistence or worsening of patients' headache may be attributed to raised intraocular pressures as opposed to lack of response to therapy.

Topiramate induced angle closure is an indiosyncratic reaction and can occur in otherwise normal eyes with normal anterior chamber angles. The management of topiramate related acute angle closure glaucoma requires cessation of the drug in concert with the primary physician and use of topical and oral aqueous suppressants. Use of pilocarpine can lead to further narrowing of the angles and worsening of signs and symptoms. Peripheral iridotomy, a traditional treatment for angle closure glaucoma, may not be of value as precipitating mechanism is not pupillary block. Topical cycloplegic agents can be given as they lower intraocular pressures by retracting the cilliary process. [3]

Topiramate induced angle closure glaucoma and transient myopia usually resolves with discontinuation of the drug. Visual outcome is usually good and episodes resolve within few weeks. In addition to its use in epilepsy, topiramate has demonstrated efficacy in other diseases and disciplines. This report highlights the need for a high index of suspicion when dealing with acute angle closure glaucoma in patients using topiramate, as this condition is reversible and treatment is typically supportive. Thus, patients should be cautioned about this potential side-effect, and instructed to seek attention should they develop blurred vision and/or eye pain following initiation or dose escalation of topiramate.

## Abbreviations

IOP – Intraocular Pressure

OD – Ocular Dexter

OS – Ocular Sinister

OU – Ocular Utrique

BCVA – Best-corrected visual acuity

## Competing interests

The author(s) declare that they have no competing interests.

## Authors' contributions

KVC and TT identified the case and directly participated in management. They also revised the manuscript and verified its intellectual content.

FS, SA, and VSB worked in collaboration to collect data, acquire clinical photographs, and draft, revise, and reference the manuscript.

All authors read and approved the final manuscript

## Consent

Written informed consent was obtained from the patient for publication of this case report and any accompanying images. A copy of the written consent is available for review by the Editor-in-Chief of this journal.
